# A Federated Adversarial Fault Diagnosis Method Driven by Fault Information Discrepancy

**DOI:** 10.3390/e26090718

**Published:** 2024-08-23

**Authors:** Jiechen Sun, Funa Zhou, Jie Chen, Chaoge Wang, Xiong Hu, Tianzhen Wang

**Affiliations:** School of Logistic Engineering, Shanghai Maritime University, Shanghai 201306, China

**Keywords:** fault diagnosis, federated learning, information discrepancy, federated adversarial

## Abstract

Federated learning (FL) facilitates the collaborative optimization of fault diagnosis models across multiple clients. However, the performance of the global model in the federated center is contingent upon the effectiveness of the local models. Low-quality local models participating in the federation can result in negative transfer within the FL framework. Traditional regularization-based FL methods can partially mitigate the performance disparity between local models. Nevertheless, they do not adequately address the inconsistency in model optimization directions caused by variations in fault information distribution under different working conditions, thereby diminishing the applicability of the global model. This paper proposes a federated adversarial fault diagnosis method driven by fault information discrepancy (FedAdv_ID) to address the challenge of constructing an optimal global model under multiple working conditions. A consistency evaluation metric is introduced to quantify the discrepancy between local and global average fault information, guiding the federated adversarial training mechanism between clients and the federated center to minimize feature discrepancy across clients. In addition, an optimal aggregation strategy is developed based on the information discrepancies among different clients, which adaptively learns the aggregation weights and model parameters needed to reduce global feature discrepancy, ultimately yielding an optimal global model. Experiments conducted on benchmark and real-world motor-bearing datasets demonstrate that FedAdv_ID achieves a fault diagnosis accuracy of 93.09% under various motor operating conditions, outperforming model regularization-based FL methods by 17.89%.

## 1. Introduction

As the core driving components of port cranes, motors operate under complex and variable conditions for extended periods, inevitably facing significant risks of fatigue, wear, and failure [1]. The health status of rolling bearings, which are critical load-bearing components, directly affects the safe operation of these motors. However, the limited working conditions and fault types in a single crane, coupled with significant disparities in data distribution across various conditions, severely impact the optimization of fault diagnosis models [2]. Therefore, developing a fault diagnosis model capable of addressing multiple fault classes under complex working conditions within an FL framework is essential for the effective health management of port cranes.

Existing fault diagnosis methods can generally be categorized into three approaches: signal-based analysis; fault mechanism-based modeling; and data-driven methods [3]. Signal-based fault diagnosis methods identify fault types by extracting sparse representations of faults from monitoring data. However, this approach relies heavily on expert knowledge, making the diagnostic results somewhat subjective. Fault mechanism-based modeling methods, on the other hand, struggle to cope with time-varying nonlinear systems [4]. As a prominent data-driven approach, deep learning (DL), with its powerful nonlinear adaptation capabilities, has been widely employed to process and analyze operational state data to extract fault features. [5,6]. In the area of early fault detection, scholars have developed methods based on stochastic resonance analysis to extract weak fault characteristics from noisy signals for diagnosing early-stage faults in rotating machinery. Additionally, a multi-objective optimization coupled neuron model has been proposed for the identification of early mechanical faults [7,8,9]. For fault diagnosis under multiple working conditions, domain adaptive cross-condition diagnostic models have been developed [10,11]. To address the challenge of diagnosing missing fault types, researchers have developed methods for generating missing type samples and global fault models based on federated learning [12,13]. However, the effectiveness of DL models is highly dependent on large amounts of high-quality and identically distributed (i.i.d.) data. In practice, port cranes typically operate most of their lifetime, resulting in a significantly low proportion of fault data [14]. In addition, since hoist motors typically operate under variable speed and load conditions, acquiring high-quality i.i.d. data becomes very challenging [15]. Data from different operating conditions often exhibit significant distributional disparities, severely compromising the feature extraction capabilities of DL models. As a result, individual clients struggle to train robust fault diagnosis models using limited fault samples from limited working conditions, rendering them ineffective in dealing with potentially unknown working conditions and new fault classes.

FL is a collaborative training mechanism that facilitates the complementation and sharing of information across clients [16]. By fully integrating fault data of various classes and from different working conditions across clients, it is possible to establish a robust global fault diagnosis model. Traditional FL methods effectively alleviate the issue of insufficient fault samples by incorporating collaborative training from multiple clients, thereby improving the fault diagnosis performance of the models [17,18,19]. However, due to the frequent and staged variations in the hoisting speeds of port cranes, motors often experience frequent transitions between start and stop states, resulting in significant disparities in motor working conditions across different clients. Consequently, the fault data collected from crane equipment sensors at different clients exhibit significant distribution heterogeneity. This heterogeneity leads to deviations in the optimization directions of local fault models at each client, ultimately affecting the generalization performance of the global model. Furthermore, it is challenging for a single client to experience and collect data on all possible fault types within the service life of its motors [20]. Therefore, during the model training process, the lack of data for certain fault types may result in the trained model having inadequate recognition capabilities for those fault classes, affecting fault diagnosis accuracy.

Existing improved algorithms based on FL primarily focus on devising various aggregation strategies to improve the quality of global models [21,22,23]. While these methods alleviate some of the challenges posed by multi-condition problems, they do not fundamentally address the intrinsic differences in feature distributions across clients. As a result, global models often struggle to adapt to clients with complex data distributions, particularly in scenarios with limited data samples and complicated multi-condition problems, where existing metrics frequently lack the accuracy needed to measure discrepancies between clients. In FL for fault diagnosis modeling, local models from clients with heterogeneous statistical distributions often exhibit inconsistent optimization directions. This statistical distribution heterogeneity prevents global models from meeting the optimization objectives of individual clients, leading to locally optimal solutions that not only degrade the performance of fault models but may also induce negative transfer phenomena [24]. Current approaches to mitigating statistical distribution heterogeneity primarily address the issue from the perspective of model drift, overlooking the fact that deep-seated discrepancies in fault information between different clients are the root cause of statistical distribution heterogeneity [25,26,27]. These discrepancies can result in inconsistent optimization directions between local models, ultimately causing misalignment between the optimization of global and local models.

This study proposes an innovative FedAdv_ID method. This method integrates a consistency evaluation metric into the local model training process to quantify the discrepancies between drifting local and global average fault information. By establishing an adversarial training mechanism between clients and the federated center, this method aims to eliminate information disparities, ensuring alignment between local optimization directions and the global model optimization direction, thereby significantly reducing the probability of negative transfer phenomena. In addition, a loss function-based optimal aggregation weight learning strategy is designed at the federated center to enhance the generalization capability of the global model. The main contributions of this study are as follows:

1. A federated adversarial fault diagnosis framework driven by information discrepancy is proposed, incorporating a feature consistency evaluation metric to quantify the feature distribution discrepancies between local and global models;
2. A probability distribution consistency loss function is developed to facilitate adversarial training between client parameters and the federated central model parameters. This approach aims to eliminate distribution discrepancies among clients and addresses the issue of negative transfer in federated learning;
3. An optimal model aggregation strategy driven by fault information discrepancy is designed to guide the construction of the global model, enhancing its generalization capability;
4. The proposed method demonstrates robust fault diagnosis performance when evaluated on benchmark and real-world datasets under various working conditions, including scenarios with incomplete fault class information.

## 2. Related Works

### 2.1. Fault Diagnosis Based on FL

FL facilitates the joint optimization and training of fault diagnosis models across multiple clients while avoiding privacy conflicts through model aggregation [28]. Chen et al. [9] proposed a federated transfer learning (FTL) framework based on difference-based weighted federated averaging (D-WFA) to collaboratively train well-trained global diagnostic models and designed an aggregation strategy using a dynamic weighted averaging algorithm with maximum mean difference (MMD) but ignored the inconsistency of the features between clients. Lu et al. [29] proposed a novel clustering-based FL algorithm. This algorithm involves training a probabilistic deep learning model, allowing each client to assess the prediction uncertainty of other clients’ models on its local dataset. Clients are then clustered based on the similarity of their datasets, thereby reducing the uncertainty across clients with varying data quality and ensuring the effectiveness of the federated process. Wang et al. [22] proposed a novel federated contrastive prototype learning scheme that facilitates collaborative modeling between the central server and multiple clients to establish a global fault diagnosis model while preserving data privacy. Mehta et al. [23] developed a duplex classifier based on 1D-CNN, which separates mixed fault classification tasks into two parallel networks for diagnosis. Zhang et al. [30] introduced a multi-scale recursive FL framework that allows the federated center to prioritize valuable client information. This framework utilizes federated center information through local multi-scale feature fusion, thereby constructing a reliable fault diagnosis model capable of handling multiple working conditions. Chen et al. [31] addressed the challenge of insufficient training data collection by individual industrial entities by incorporating capsule-based fault feature expressions into an FL framework. This approach reduces the transmission burden and enhances data security. Qin et al. [32] proposed an improved FL algorithm that reduces computational costs through wavelet packet decomposition. This algorithm employs SecureBoost to train local diagnostic models during the modeling process, aggregating model parameters from multiple railway lines in each iteration. Du et al. [33] tackled the issue of insufficient generalization capability in fault models with small samples by combining the Forgetting Kalman Filter (FKF) with Cubic Exponential Smoothing (CES) during the model aggregation process. This approach enables high-precision fault diagnosis in mixed fault scenarios across multiple clients.

Existing FL frameworks generally assume that each client is ideally isomorphic. However, different clients have diverse data distributions, and FL needs to deal with the statistical distribution heterogeneity to achieve effective joint optimization. Shi et al. [34] proposed a method that corrects the optimization direction of the model by introducing a sparse regularization term in the optimization function of the local client, thereby inhibiting the drift of the local model. This approach has proven effective in mitigating statistical distribution heterogeneity. However, this algorithm primarily focuses on eliminating discrepancies with the global model at the model level. Therefore, fully leveraging fault information, such as features from different clients under multiple working conditions, is crucial for enhancing the effectiveness of FL-based fault diagnosis in the presence of statistical data distribution heterogeneity.

### 2.2. Federated Heterogeneity Elimination under Multiple Working Conditions

Existing FL approaches are typically designed for joint optimization under the assumption that all clients operate under ideal homogeneous conditions. However, motors at different client locations function under diverse operational environments, which can significantly impact model optimization across clients and adversely affect the generalization performance of the global model.

Li et al. [35] addressed feature skew in statistical heterogeneity by adding batch normalization layers to local models, but the effectiveness of this method in handling more complex heterogeneity, such as quantity skew, remains uncertain. Yoon et al. [36] proposed a global Mixup method that allows clients to access averaged data from other clients, optimizing their data distribution to mitigate the non-i.i.d issue. Wang et al. [37] proposed a general framework for analyzing the convergence of federated heterogeneous optimization algorithms. They provided a first-principle understanding of solution bias and the slowdown of convergence due to objective inconsistencies, which can eliminate discrepancies in the presence of local quantities. Chen et al. [38] proposed a dynamically weighted federated aggregation strategy that uses the Manhattan distance to measure the discrepancy between local and global models as the basis for weight aggregation, effectively reducing the impact of poor local data. However, the Manhattan distance may fail to converge when a client’s data distribution is complex under multiple working conditions. To address the non-i.i.d. data heterogeneity problem, Chen et al. [39] proposed a synthetic data generation method. This approach employs knowledge distillation to learn from heterogeneous models of participating clients, facilitating model aggregation at the central server. Wang et al. [40] proposed a dynamic model aggregation algorithm to mitigate the impact of low-quality clients by evaluating each client’s weight based on its contribution to the total consensus diagnostic knowledge and then aggregating local models using adaptive weights. However, this approach performs poorly when confronted with unknown and complex operational tasks due to lacking more informative data.

Current strategies for mitigating federated heterogeneity under multiple working conditions primarily focus on two approaches: regularization terms and transfer learning. Regularization terms address heterogeneity solely from a model perspective, leading to significantly compromised diagnostic performance when confronted with data from newly emerging fault classes. Conversely, transfer learning methods aim to align feature distributions across different working conditions at the feature level; similarity clustering of client performance under a certain dataset can only be used for client performance discrimination and cannot be personalized for a specific low-quality client. However, their diagnostic performance exhibits considerable variability. These existing methods show limitations when dealing with complex multi-condition FL scenarios.

## 3. Federated Adversarial Fault Diagnosis Methodology Driven by Information Discrepancy

Step 1: Federated adversarial fault diagnosis framework driven by information discrepancy

The accuracy of high-dimensional fault features and probability density distributions extracted by various clients directly influences the performance of their diagnostic models. However, due to the heterogeneity of feature distributions across clients, this fault information can undergo significant drift, making the global model misaligned with the optimization direction of local training. This misalignment ultimately reduces fault diagnosis accuracy for each client. Existing methods primarily address the issue of global model bias caused by inconsistent optimization directions among clients from the perspective of model drift. However, these approaches fail to fully exploit the fault information shared among clients and overlook the impact of fault information drift on the generalization capability of the global model.

This section establishes the FedAdv_ID framework between clients and the federated center. The framework includes metrics for measuring global fault information and fault information discrepancies, a federated adversarial training mechanism to address statistical distribution inconsistencies between clients, and a federated aggregation strategy driven by fault information discrepancy. This design ensures that the federated center can utilize client information both fairly and efficiently.

**Figure 1:**
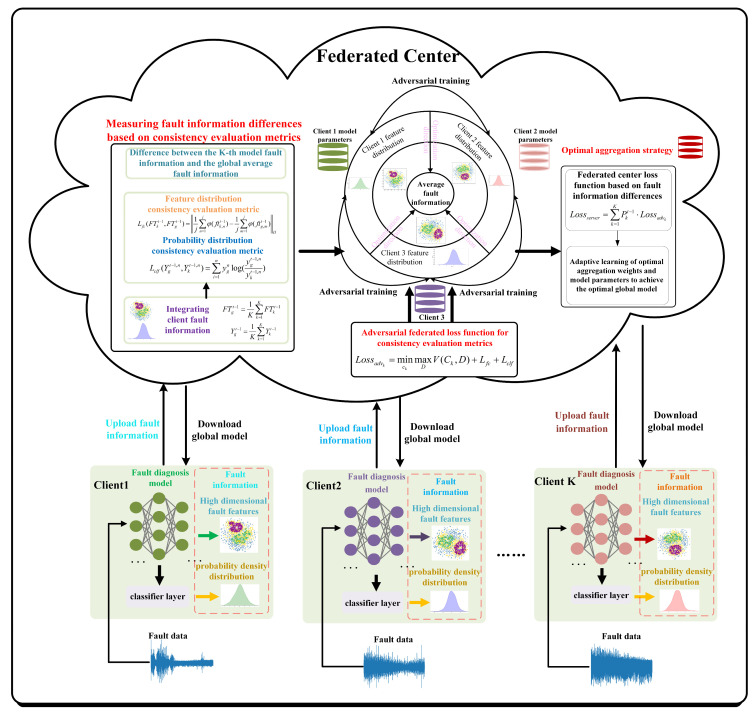
Schematic diagram of the federated adversarial method driven by information discrepancy.

Specifically, the federated center integrates client fault information to derive the global average fault information. A consistency evaluation metric is designed based on the distance between the client and global average fault information. This metric guides the federated adversarial training and is the updated direction for the client models to reduce discrepancies between local and federated fault information. After each round of federated adversarial training, the discrepancies in the consistency evaluation metric between local and global average fault information are used to assign aggregation weights to different client models. This approach ensures that rare fault information from lower-quality clients operating under various conditions is effectively incorporated into the federated diagnosis model. The schematic diagram of the FedAdv_ID method is shown in Figure 1, and implementation details are provided in the following steps.

Step 2: Federated global fault information metric

During the t−1 round of communication, the federated center initializes the federated discriminator model parameter $\theta_{d}^{t-1}$ and the global model $\theta_{g}^{t-1}$, which are then distributed to each client. Upon downloading the global model, each client uses locally available bearing data with inconsistent statistical distributions ${(X}_{i},i=1,2,\ldots n)$ to train their local model parameters $\theta_{i}^{t-1}$. The feature extraction process for the clients is described in Equation (1).

(1) $$FT_{k}^{t-1}=F(\theta_{g}^{t-1},X_{i}),i\in 1,2,\ldots ,n$$

where *k* denotes the client index, and ${FT}_{k}^{t-1}$ denotes the high-dimensional fault features extracted by the *k*-th client after the t−1 round of communication, F(·) denotes Feature extraction networks. Subsequently, these high-dimensional features are input into the classifier layer to obtain the probability distribution of different fault classes, as shown in Equation (2).

(2) $$Y_{k}^{t-1}=\frac{\exp(w_{j}FT_{k}^{t-1})}{\sum_{j=1}^{c}\exp(w_{j}FT_{k}^{t-1})},k\in 1,2,\ldots ,K$$

where $Y_{k}^{t-1}$ denotes the probability distribution of the fault samples belonging to different fault categories for client *k*; *c* denotes fault classes.

The clients then upload the fault information and the corresponding classes probability distribution ${(FT}_{k}^{t-1},Y_{k}^{t-1})$ to the federated center. The federated center integrates fault information from different clients and computes the average to form a global fault information set, as shown in Equations (3) and (4).

(3) $$FT_{g}^{t-1}=\frac{1}{K}\sum_{k=1}^{K}FT_{k}^{t-1}$$

(4) $$Y_{g}^{t-1}=\frac{1}{K}\sum_{k=1}^{K}Y_{k}^{t-1}$$

where K denotes the total number of clients participating in the federation. ${FT}_{g}^{t-1}$ and $Y_{g}^{t-1}$ denote the average fault feature information and average classification probability information at the federation center, respectively. The new fault information set encompasses the average fault information from all clients, effectively addressing the issues of insufficient richness in local fault features and low probability density entropy. Furthermore, the average fault information serves as the optimal direction for updating fault information in the local models toward the global model, guiding client models to update in a direction that reduces information discrepancies.

This approach results in an optimal global fault diagnosis model that integrates fault information from various working conditions. It is important to note that when discrepancies exist in the number of fault samples across clients, batch gradient descent optimization or the expansion of output features and their corresponding probability distributions is necessary to ensure the accurate calculation of the federated center’s average fault information. The process of expanding the fault information from clients is detailed in Equations (5) and (6).

(5) $$FT_{k}^{t-1}=Broadcast[FT_{k}^{t-1},0]$$

(6) $$Y_{k}^{t-1}=Broadcast[Y_{k}^{t-1},0]$$

Step 3: Consistency evaluation metric for fault information discrepancy

The participating federated clients possess only limited local working conditions and fault information, reflecting the localized characteristics of fault samples. In contrast, the federated center’s global fault information integrates fault data from diverse working conditions. Therefore, a consistency evaluation metric based on Step 2 is used to measure the discrepancies between client-specific fault information and the federated global fault information. This metric guides the adversarial federated training process, aiming to reduce inconsistencies between local and global fault information. Consequently, the optimization directions of the client model parameters become more aligned, ensuring the effective aggregation of the federated model. The construction process of the consistency evaluation metric is detailed in Equations (7) and (8).

(7) $$L_{fc}(FT_{k}^{t-1},FT_{g}^{t-1})={\left\|\frac{1}{j}\sum_{n=1}^{j}\varphi (ft_{k,n}^{t-1})-\frac{1}{j}\sum_{m=1}^{j}\varphi (ft_{g,m}^{t-1})\right\|}_{H}$$

(8) $$L_{clf}(Y_{g}^{t-1,n},Y_{k}^{t-1,n})=\sum_{i=1}^{n}y_{g}^{n}\log(\frac{y_{g}^{t-1,n}}{y_{k}^{t-1,n}})$$

where $L_{fc}$ and $L_{clf}$ denote the feature consistency evaluation metric and the probability consistency evaluation metric, respectively. φ(·) denotes the mapping to the Reproducing Kernel Hilbert Space (RKHS) of the features [41]. ${ft}_{k,n}^{t-1}$ denotes the high-dimensional features of the *n*-th sample from client *k* in the *t*−1 communication round, and $y_{k}^{t-1}$ denotes the probability distribution of fault categories for the *n*-th sample from client *k* in the (*t* − 1) communication round.

The metrics $L_{fc}$ and $L_{clf}$ quantify the discrepancies between local fault information and the average fault information. These metrics guide the optimization directions of feature extractors and classifiers across clients, thereby mitigating the issue of fault information drift caused by statistical distribution heterogeneity.

Step 4: Federated adversarial optimization for feature inconsistencies between clients

**Figure 2:**
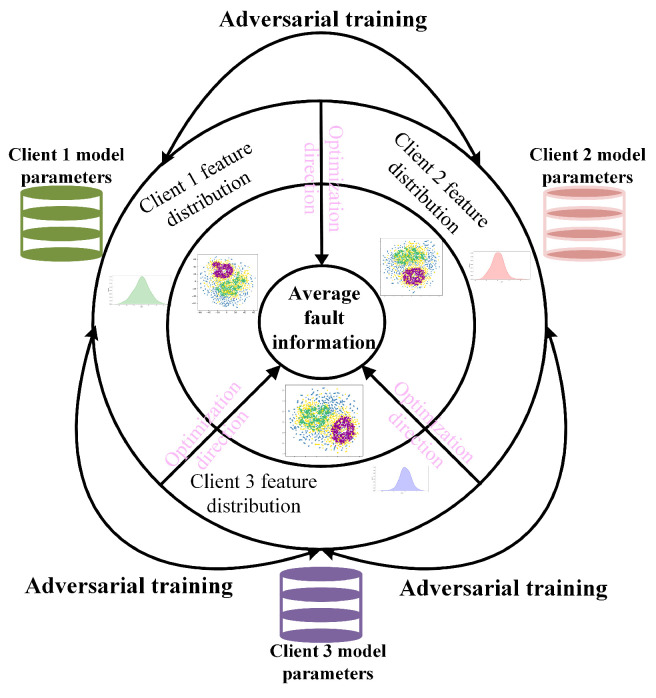
Eliminate discrepancy between fault information.

The statistical distribution heterogeneity of local client fault data leads to a drift in the fault information of clients. To mitigate the negative impact of this drift on model optimization, it is crucial to eliminate the discrepancies between the clients’ fault information and the federated center’s average fault information. To this end, a federated adversarial training mechanism is designed to address statistical distribution inconsistencies among clients, thereby reducing the discrepancies between their fault information and that of the federated center. The schematic diagram of the algorithm designed to eliminate these discrepancies is presented in Figure 2.

The purpose of federated adversarial training is to eliminate discrepancies in features and probability distributions across different working conditions through a max-min game between local fault information and global average fault information. This process enhances the generalization capability of the global model. By incorporating the consistency evaluation metric as a regularization term in the adversarial loss function, the model penalizes fault information drift caused by the statistical distribution heterogeneity of local clients. This approach reduces the extent of fault information drift and improves the accuracy of high-dimensional fault features extracted by the feature extractor, as well as the probability density distribution estimated by the classifier. The value function for adversarial federated training is provided in Equations (9) and (10).

(9) $${\mathrm{Loss}}_{\mathrm{adv}}=\underset{c_{k}}{\min}\underset{D}{\max}V(C_{k},D)+L_{fc}+L_{clf}$$

(10) $$\underset{c_{k}}{\min}\underset{D}{\max}V(C_{k},D)=E_{FT_{g}^{t-1}\sim P_{FT_{g}^{t-1}}}[\log D(FT_{g}^{t-1})]+E_{FT_{k}^{t-1}\sim P_{FT_{k}^{t-1}}}[\log(1-D(FT_{k}^{t-1}))]$$

where $C_{k}$ denotes the client generator, and D denotes the federated center discriminator. $L_{fc}$ denotes fault feature consistency regularization term; $L_{clf}$ denotes fault information probability distribution consistency regularization term. ${FT}_{g}^{t-1}\sim P_{{FT}_{g}^{t-1}}$ denotes a sample feature that follows the distribution of the average fault features, while ${FT}_{k}^{t-1}\sim P_{{FT}_{k}^{t-1}}$ denotes the fault feature distribution of a sample from client *k*. During adversarial training, the clients minimize the loss function in Equation (9) to optimize the generator parameters, continuously reducing the discrepancies between fault features. The federated center’s discriminator maximizes the adversarial loss function to optimize its parameters. The optimization directions of the two parts are entirely opposite, forming a minimax adversarial game between the “two players,” ultimately reaching a steady-state Nash equilibrium [42].

It is important to note that the adversarial loss function in Equation (9) can experience significant instability during the optimization of generator and discriminator parameters due to differences in fault information among clients. Therefore, adding consistency metrics to the adversarial loss function can guide the fault features extracted by each client to align with the federated center’s average fault features, ensuring the consistency of the optimization direction of the client generators and thereby reducing the instability in the optimization of generator parameters. Additionally, the optimized generator-extracted features can better deceive the discriminator, ensuring the stability of the discriminator’s optimization process. However, when there are significant differences in fault features among clients, the quality of the federated center’s average fault feature information may be affected. To ensure the stability of the generative adversarial process, new regularization terms need to be designed to penalize the imbalance in the optimization process. Furthermore, for clients with poor fault information quality, it is necessary to set a threshold for their adversarial loss to reduce fluctuations in training loss. By introducing new gradient penalty terms into Equation (9), potential instability in adversarial training can be addressed.

(11) $${\mathrm{Loss}}_{advk}=\underset{c_{k}}{\min}\underset{D}{\max}V(C_{k},D)+L_{fc}+L_{clf}+\gamma E_{FT_{k}^{t-1}\sim P_{FT_{k}^{t-1}}}{\left({\left\|\nabla_{FT_{k}^{t-1}}D(FT_{k}^{t-1})\right\|}_{2}-1\right)}^{2}$$

where ${\left(E_{{FT}_{k}^{t-1}\sim P_{{FT}_{k}^{t-1}}}{\left\|\nabla_{{FT}_{k}^{t-1}}D\left({FT}_{k}^{t-1}\right)\right\|}_{2}-1\right)}^{2}$ denotes the gradient penalty term; γ denotes the gradient penalty term coefficient.

Due to the significant statistical distribution differences in the fault data used by clients for training, minimizing $V\left(C_{k},D\right)$ would increase the confidence of the federated center’s discriminator, thereby rejecting new samples generated by clients through local data and adversarial training. This outcome is counterproductive to the goal of enabling local clients to learn the global average fault features and reduce distribution discrepancies. Therefore, this study opts to have clients maximize the loss function while the central server’s discriminator minimizes the loss function, creating a game between the local clients and the central server’s discriminator. This approach provides stronger gradients for early adversarial training. The process of backpropagating the adversarial training loss function ${Loss}_{adv}$ based on the consistency evaluation metric to optimize the parameters of the federated discriminator model and the client generator models is detailed in Equation (12).

(12) $$\{\begin{array}{c} \theta_{d}^{t}=\theta_{d}^{t-1}-lr\cdot \frac{\partial Loss_{advd}}{\partial \theta_{d}^{t-1}}\\ \begin{array}{c} \theta_{1}^{t}=\theta_{1}^{t-1}-lr\cdot \frac{\partial Loss_{adv1}}{\partial \theta_{1}^{t-1}}\\ \theta_{2}^{t}=\theta_{2}^{t-1}-lr\cdot \frac{\partial Loss_{adv2}}{\partial \theta_{2}^{t-1}}\\ \vdots \\ \theta_{k}^{t}=\theta_{k}^{t-1}-lr\cdot \frac{\partial Loss_{advk}}{\partial \theta_{k}^{t-1}} \end{array} \end{array}$$

where $\theta_{d}^{t}$ denotes the updated parameters of the federated discriminator; $\theta_{k}^{t-1}$ denotes the backpropagation-optimized model parameters of the *k*-th client, and *lr* is the learning rate. Equation (12) demonstrates that when each client’s generator engages in adversarial training with the federated discriminator, both sets of parameters are updated simultaneously. Through multiple rounds of adversarial training, the federated discriminator optimizes its parameters and enhances its discriminative ability. This process encourages clients to actively improve their local models’ learning of average fault information.

It is important to note that because the optimization directions of the discriminator and the client’s generator are opposite, the adversarial training error will decrease but eventually converge to a Nash equilibrium point. Let $Loss_{c_{k}}=\underset{c_{k}}{\min}V(C_{k},D)$ denote the loss function of the client and $Loss_{d}=\underset{d}{\max}V(C_{k},D)$ denote the loss function of the discriminator. The equilibrium point obtained through adversarial training is given by Equation (13).

(13) $$\lim_{t\to \infty }\left|{\mathrm{Loss}}_{c_{k}}-{\mathrm{Loss}}_{d}\right|=N,N\in R^{+}$$

where *N* denotes the Nash equilibrium point at which the client generator and the federated discriminator can no longer update through adversarial training.

When ${Loss}_{adv}$ continually decreases and reaches a predefined threshold, it indicates that the clients have been effectively optimized through adversarial training, and the discrepancies between local fault information and aggregate fault information are minimized. This process mitigates the fault information drift caused by statistical distribution heterogeneity. Through federated adversarial training among clients, local fault information becomes initially adaptable to the global fault diagnosis model. However, the performance of this global model in local fault diagnosis tasks is contingent upon the soundness and reliability of the global model aggregation strategy. To address this, an information discrepancy-driven federated aggregation strategy has been developed to ensure that the federated center achieves an optimal global diagnosis model.

Step 5 Effective recognition of fault features by clients

In step 4, by measuring the discrepancy between each client’s fault information and the federated center’s average fault information, adversarial optimization effectively resolves the inconsistency in model optimization directions caused by differences in operating conditions between clients. This approach ensures that the federated diagnosis model can generalize fault information across different client distributions. However, the diagnostic accuracy of the federated model relies heavily on the training effectiveness of the client models. Therefore, while eliminating the discrepancies in fault information among clients, it is also essential to further analyze the fault information inconsistency metrics and the guidance provided by adversarial loss during the client model training process. This ensures that the local models can effectively recognize fault features.

The local client model training process should not only include a fixed cross-entropy loss to achieve fault classification accuracy on private datasets but also incorporate an adversarial loss function to address fault information discrepancies. This optimizes the feature extraction network to progressively reduce fault feature differences among clients. Additionally, to better measure the discrepancy between client fault features and the federated center’s average features, a feature discrepancy regularization term needs to be added during the client training process to ensure consistent optimization direction of the local model. The collaborative optimization of these three loss functions ensures that the local model can effectively extract well-represented fault features while further reducing fault information discrepancies among clients. It should be noted that the fault information discrepancy regularization term is already included in the adversarial loss. Therefore, the training loss of the local model can be expressed as

(14) $${Loss}_{clientk}=Loss_{advk}+\left(-\sum_{c_{i}}^{C}y_{c_{i}}\log({\hat{y}}_{c_{i}})\right)$$

where C denotes fault type; $y_{c_{i}}$ denotes the true label of the sample, and ${\hat{y}}_{c_{i}}$ denotes the predictive label of the sample.

As described in Equation (14), the parameter optimization process of the local model in federated learning follows the formulation outlined in Equation (15).

(15) $$\{\begin{array}{c} \theta_{1}^{t,n}=\theta_{1}^{t,n-1}-lr^{\prime }\cdot \frac{\partial Loss_{client1}}{\partial \theta_{1}^{t,n-1}}\\ \theta_{2}^{t,n}=\theta_{2}^{t,n-1}-lr^{\prime }\cdot \frac{\partial Loss_{client2}}{\partial \theta_{2}^{t,n-1}}\\ \vdots \\ \theta_{k}^{t,n}=\theta_{k}^{t,n-1}-lr^{\prime }\cdot \frac{\partial Loss_{clientk}}{\partial \theta_{k}^{t,n-1}} \end{array}$$

where $\theta_{k}^{t,n}$ denotes the parameters of the *k*-th client after the *n*-th iteration of optimization in the *t*-th round of the federated process, and ${lr}^{\prime }$ denotes the learning rate for local model optimization.

In this process, the adversarial loss is generated at the federated center and then transmitted to the client, where it works in conjunction with the cross-entropy loss function to optimize the model parameters. This approach does not impede the real-time updating of local model parameters, as the adversarial loss does not require multiple iterations at the federated center. Instead, once the global model is received, the local model undergoes multiple iterative updates on the client’s private data. Consequently, both the global model and the adversarial loss can be delivered simultaneously to the client, facilitating the optimization of the local model.

Step 6: Federated aggregation strategy driven by fault information discrepancy

The information discrepancy between different clients varies, and the federated center should account for this when aggregating local models by assigning weights accordingly. Clients whose local fault information significantly deviates from the average fault information tend to perform poorly in adversarial training and should be assigned less weight. Conversely, clients with smaller discrepancies perform better and should be assigned greater weight. In this section, we propose a federated aggregation strategy driven by fault information discrepancies, where the weights are linked to the federated adversarial loss function. This approach ensures that the allocation of aggregation weights is determined solely by the discrepancy between the client and global fault information, as illustrated in Equation (16).

(16) $${\mathrm{Loss}}_{\mathrm{sever}}=\sum_{k=1}^{K}P_{k}^{t-1}\cdot {\mathrm{Loss}}_{adV_{k}}$$

where $P_{k}^{t-1}$ denotes the aggregation weight of the *k*-th client in the *t*−1 round of the federation. The adversarial loss function adaptively learns the aggregation weights based on the client discrepancies, reducing the overall inconsistency in fault information to obtain the optimal global model. The optimization process for the client generator and aggregation weights is detailed in Equation (17).

(17) $$\{\begin{array}{c} P_{k}^{t-1}=P_{k}^{t-1}-lr\cdot \frac{\partial Loss_{server}}{\partial p_{k}^{t-1}}\\ \theta_{k}^{t-1}=\theta_{k}^{t-1}-lr\cdot \frac{\partial Loss_{server}}{\partial \theta_{k}^{t-1}} \end{array}$$

The global fault model aggregation process for the new federated round is shown in Equation (18) derived from Equation (17).

(18) $$\theta_{g}^{t}=\sum_{k=1}^{K}P_{k}^{t-1}\cdot \theta_{k}^{t-1}$$

In the new aggregation round, the federated center’s loss function is constructed based on this discrepancy, and it adaptively learns and updates the optimal aggregation weights and model parameters according to this loss function. As a result, it is distributed to each local client for a new round of information discrepancy-driven adversarial federated fault model training.

The dynamic interaction between clients and the federated center regarding fault information and model parameters implies that a client’s fault feature distribution evolves with changing operating conditions. In response, the client extracts new fault features using the generator and uploads them to the federated center, thereby updating the federated center’s average fault information. As a result, the consistency evaluation metric between the client’s fault features and the average fault features is adjusted, leading to a new adversarial loss that optimizes the parameters of both the federated center’s discriminator and the client’s generator. Changes in the adversarial loss value clearly reflect the quality of fault features across clients and the effectiveness of their local models. Therefore, it is essential to fully consider the data quality attributes among clients during the federated model aggregation process. At this stage, we introduce a learnable parameter $P_{k}$ that adapts based on changes in the adversarial loss value between the client’s fault features and those of the federated center. This mechanism ensures that clients with varying data quality are assigned weight parameters that correspond to their data quality during model aggregation. Consequently, the federated model can dynamically adapt to the continuously evolving data distributions across clients, thereby enhancing its generalization capability.

### Fault Diagnosis Based on FedAdv_ID

**Figure 3:**
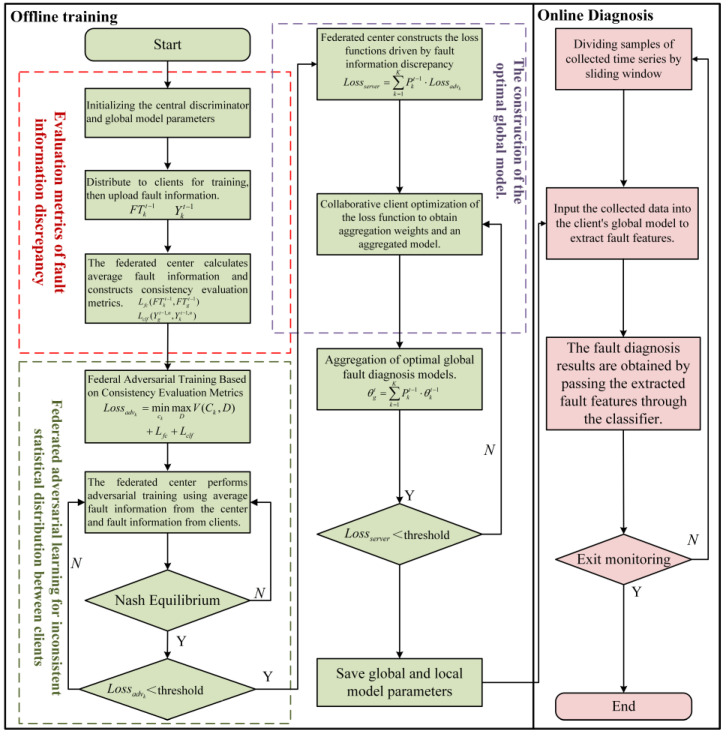
Flowchart of the FedAdv_ID method.

The implementation process of the FedAdv_ID method comprises two parts: offline training and online diagnosis. The proposed algorithm trains the fault diagnosis model using multiple working condition data within the client during the offline training stage. The training process culminates when the loss function at the federated center achieves a predefined threshold, at which point both the global and local model parameters are saved. Subsequently, each client utilizes its local model to extract fault features from online monitor data, thereby diagnosing the motor’s fault state at the current moment, as illustrated in Figure 3.

The objective of adversarial training in FedAdv_ID is to identify consistent fault features by reducing discrepancies in fault characteristics among clients rather than merely suppressing the differences in fault features. The underlying principle of this adversarial mechanism is to guide the updating of client generator parameters by addressing inconsistencies between features, thereby extracting new and relevant feature information. Essentially, the overall amount of fault feature information is not diminished; instead, the critical information is preserved while the heterogeneity of fault features across clients is progressively reduced. This process indirectly enhances the representation of useful fault features. However, in this context, useful fault features are not necessarily given greater weight; rather, the degree of suppression of useful features is significantly lessened compared to that of heterogeneous features during the adversarial training process. Furthermore, the federated aggregation strategy, driven by fault information discrepancies, fully accounts for the data quality among clients, effectively mitigating the negative transfer effects caused by low-quality clients within the federated model. As a result, through the synergistic collaboration of these steps, FedAdv_ID primarily focuses on suppressing irrelevant fault information across different clients. While some useful information may inevitably be affected during this process, the impact is minimal compared to the suppression of irrelevant fault information.

## 4. Experiments and Analysis

The Case Western Reserve University (CWRU) bearing dataset [43] and the Shanghai Maritime University (SMU) electric motor fault simulation experimental platform are utilized as fault data sources to evaluate the performance of the proposed method under varying statistical distributions within FL. The experimental design encompasses a range of scenarios and employs accuracy metrics to assess the method’s performance across diverse conditions. Furthermore, a comparative analysis with several state-of-the-art methods is conducted to validate the superiority of the proposed approach.

### 4.1. Experimental Design and Analysis of the Motor Bearing Benchmark Dataset

**Figure 4:**
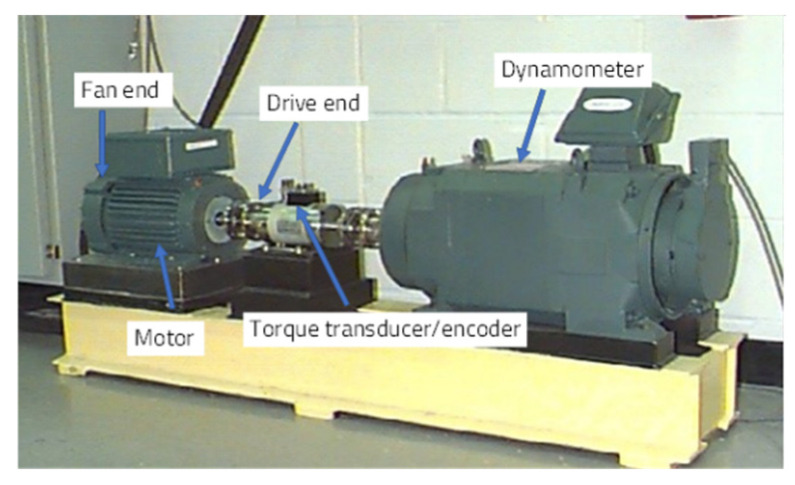
The CWRU bearing experiment bench [43].

The CWRU motor bearing platform is shown in Figure 4. Vibration acceleration signals are collected by sensors installed at both the drive and fan ends of the motor. The sampling frequencies are 12 kHz and 48 kHz at the drive end and 12 kHz at the fan end. The motor shaft, supported by the test bearing, operates under four load conditions: 0 HP; 1 HP; 2 HP; and 3 HP, corresponding to approximate motor speeds of 1797 rpm, 1772 rpm, 1750 rpm, and 1730 rpm, respectively.

**Figure 5:**
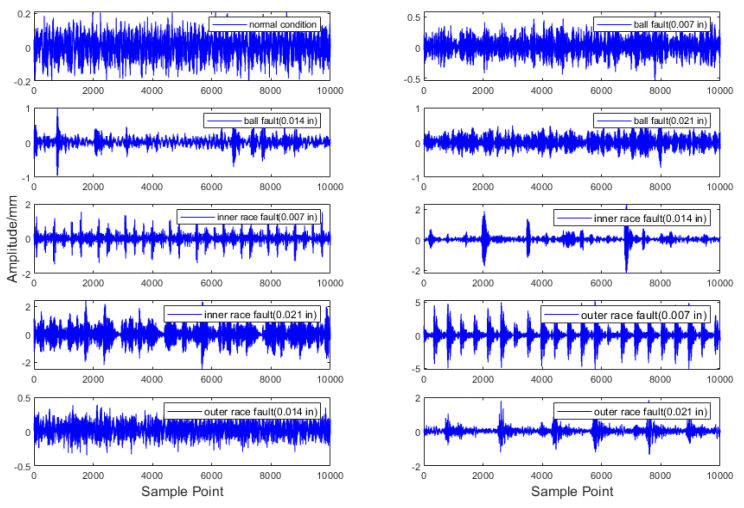
Vibration signal waveforms of ten types of bearing data.

The fault data in this section are derived from vibration signals collected by sensors at a sampling frequency of 48 kHz at the motor drive end. The dataset comprises four health states: one normal state and three fault states. The fault states correspond to rolling bearing damage at three different locations: the ball; inner race; and outer race, each with a defect size of 0.007 inches. A sliding window approach, with a window length of 100 and a step size of 50, is applied to form the final dataset. The time–domain waveform diagrams for each fault type at the drive end are illustrated in Figure 5, with the corresponding labels provided in Table 1.

Four experimental scenarios were designed to validate the effectiveness of the proposed FedAdv_ID fault diagnosis method under various working conditions. Each scenario includes three client participants within the federation. Although multiple clients may be involved in real-world applications, using a limited number here demonstrates the method’s efficacy. The specific experimental details are shown in Table 2.

To minimize the impact of random variability in the experimental results, each experiment was repeated five times with different random seeds, and the average value was calculated. To verify the superiority of the proposed method, advanced FL approaches that address statistical heterogeneity were employed as comparative baselines, and these comparative experiments were conducted under identical conditions. Table 3 provides a detailed description of the comparative methods used, while Table 4 outlines the architecture of the client generator models and the associated hyperparameters for each method.

### 4.2. Experimental Results Analysis for the CWRU Dataset 

**Figure 6:**
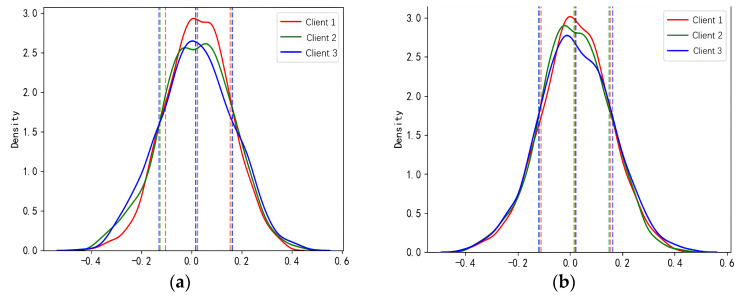
Statistical distribution of failure data under different working conditions. (**a**) Statistical distribution of fault data in experiment 1; (**b**) Statistical distribution of fault data in experiment 2.

To explore the sample distribution under different working conditions, we visualized the probability density distributions of fault samples from Experiments 1 and 2, as shown in Figure 6. In Experiment 1, all clients operated under two stable conditions, with typical fault features within each client exhibiting varying degrees of drift. In Experiment 2, all clients operated under three conditions, where the intrinsic features of the same fault type displayed diverse drift patterns. This visualization analysis provides deeper insight into how different working conditions influence the distribution of fault features.

Figure 6 displays the probability density distribution curves (solid lines) of the samples, along with their corresponding means and variances (dashed lines). In Figure 6a, Client 1’s mean converges to approximately zero, although the distribution remains uneven; Client 2 shows a distinct bimodal distribution, while Client 3 exhibits a relatively uniform distribution. Figure 6b reveals that all clients demonstrate some degree of left skewness and bimodality, reflecting the increasing statistical distribution heterogeneity. Table 5 presents the fault diagnosis results of the proposed method compared with those of the other methods under the above experimental scenarios.

The results of Experiment 1 reveal significant differences in fault diagnosis accuracy across the various methods. The DNN approach, which was unable to detect feature drift, achieved an average accuracy of only 53.80%. FedAvg improved accuracy by 7.74% through joint optimization but failed to account for the differing optimization directions among clients. FedRL_F1 introduced F1 regularization, enhancing adaptability under different statistical distributions, and improved accuracy by 13.66% compared to FedAvg. However, it did not address the impact of statistical distribution heterogeneity on error information. Fed_BN tackled feature scale disparities but did not fully resolve feature drift issues. The experiment demonstrated that larger loads and more significant drifts had a pronounced impact on fault diagnosis accuracy. WAFedL employed an adaptive weighting strategy, which improved accuracy by approximately 8% over Fed_BN but potentially neglected fault information from low-weight clients. Building on FedRL_F1, FedAdv_ID addressed information drift by incorporating fault information consistency evaluation and an adversarial federated framework. This approach effectively resolved feature drift issues while ensuring alignment between individual client optimization directions and the global model. As a result, it mitigated the potential risks associated with WAFedL’s handling of low-weight clients and addressed Fed_BN’s limitations in managing feature drift.

**Figure 7:**
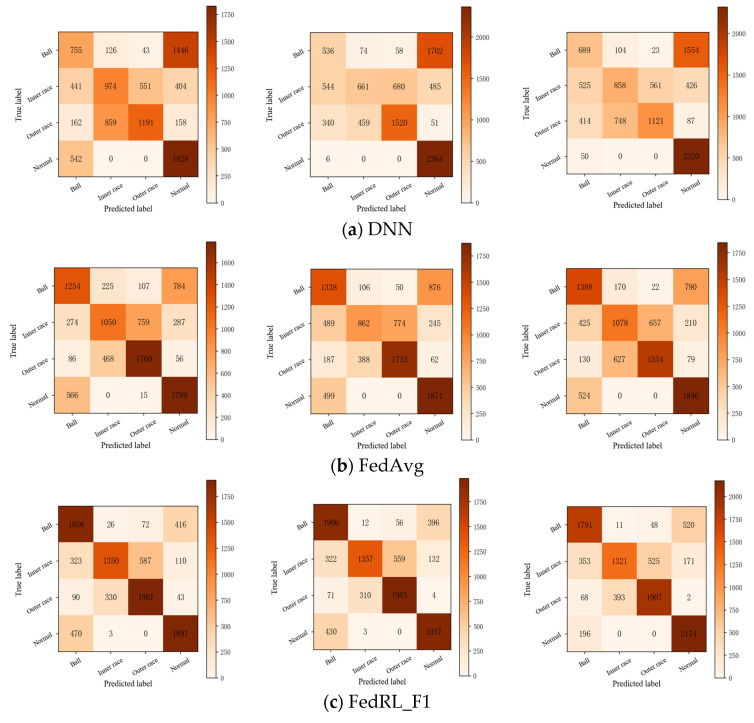

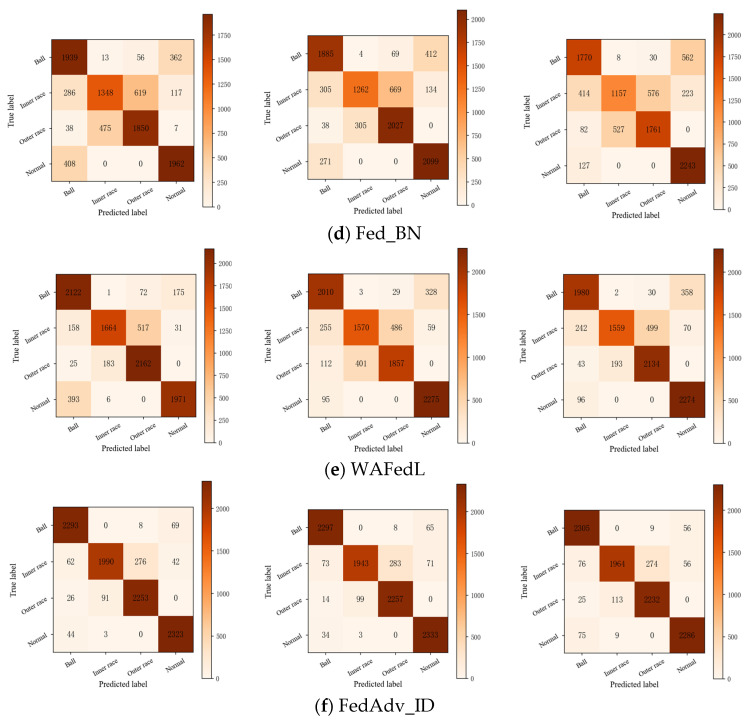
Confusion matrix of each method for Experiment 1 multiple working scenarios.

Figure 7 presents the confusion matrices for the different methods used in Experiment 1. Figure 7a illustrates that while the DNN method effectively distinguishes between normal and faulty samples, it tends to misclassify ball flaws as normal conditions. In addition, the DNN method demonstrates lower accuracy in identifying inner-race and outer-race defects compared to its performance with ball defects and normal conditions, likely due to the similarities in the defect characteristics of the inner and outer races. Figure 7b shows that FedAvg performs well in detecting outer-race faults and normal conditions, significantly improving diagnostic accuracy for ball faults; however, its performance in identifying inner-race faults remains suboptimal.

The methods depicted in Figure 7c–e demonstrate varying degrees of improvement in fault diagnostic accuracy. Notably, the ball faults and normal conditions consistently achieve the highest detection rates across most diagnostic models, although they are prone to being misclassified as each other. In contrast, inner-race and outer-race faults exhibit lower diagnostic rates and are more susceptible to cross-misclassification.

Experiment 2 introduced more complex working conditions, leading to various feature shifts within the same fault type, which posed significant challenges to the model’s feature extraction capabilities. Under these conditions, the DNN method experienced a notable 8.73% decrease in diagnostic accuracy compared to Experiment 1, as it struggled to accurately identify fault categories. FedAvg’s performance declined by 7.54% due to the increased complexity of the statistical distributions, which diminished the effectiveness of its aggregation strategy. The limitations of FedRL_F1, which primarily addresses model drift, became more pronounced under these conditions. Although it mitigated discrepancies between the model and the federation center, it failed to address the heterogeneity of data distribution, resulting in the largest accuracy drop of 13.17%. Fed_BN experienced an 11.99% decrease in accuracy, indicating that significantly shifted features continued to affect decision boundaries even when feature scaling was applied. While WAFedL experienced a relatively smaller drop in accuracy, its overall diagnostic accuracy of 75.20% suggests that its improved aggregation strategy still has limitations in optimizing specific clients. This is further evidenced by the significantly lower diagnostic accuracy of client 1 in Experiment 2 compared to Experiment 1.

In contrast, FedAdv_ID maintained the highest fault diagnostic accuracy in Experiment 2, exhibiting the smallest decrease in performance. This outcome underscores the method’s effectiveness in enhancing the generalization capability of the global model by eliminating fault information disparities and maintaining stable diagnostic performance across different experimental scenarios. Moreover, the federated aggregation strategy based on information disparity ensured alignment between the global model and local client optimization directions, facilitating improvements in local models and boosting fault diagnostic accuracy. Even under complex working conditions, FedAdv_ID achieved an impressive diagnostic accuracy of 86.06%, demonstrating its superior performance.

Experiments 3 and 4 further investigate the effectiveness of different methods in scenarios with missing fault types, building on the foundations of Experiments 1 and 2. When the failure data types differ between clients, the statistical distribution differences increase significantly (Table 6).

In Experiment 3, the DNN method that relies only on its local data for training not only suffers from low diagnostic accuracy due to multiple working conditions but also fails to learn missing fault features from other clients, resulting in further performance degradation compared to Experiment 1. Although FedAvg can learn fault features from other clients through a global model, its diagnostic effectiveness for missing faults remains suboptimal due to feature shift effects. FedRL_F1 and Fed_BN specifically address the problem of statistical distribution heterogeneity, but their ability to learn missing defect types remains limited to the global model level, yielding limited improvements. WAFedL improves the diagnostic accuracy of missing defect types by optimizing the global model, improving about 20.67% over FedAvg. However, its learning is limited to the model level. FedAdv_ID, on the other hand, generates comprehensive fault information that includes missing fault data from different clients through the federation center’s information fusion module. Using a federated adversarial framework helps eliminate inconsistencies in fault information. This approach enables learning of missing fault characteristics and overcomes the problems of insufficient fault characteristics and low probability density entropy in individual clients. As a result, it provides richer fault information for model diagnostic decision-making, thereby improving the accuracy of identifying missing fault types.

Experiment 4 builds on the complex multi-condition scenario of Experiment 2 to validate the effectiveness of the approach in extreme cases where fault types are missing. In this scenario, not only are certain fault types missing across the three conditions, but the statistical distribution heterogeneity is further exacerbated, making it impossible to effectively diagnose the missing fault types. While most methods become nearly ineffective in this context, FedAdv_ID can adaptively retrieve the missing fault information from other clients through fault feature discrepancy measurement, assigning greater weight parameters to ensure that the aggregated model can diagnose the missing faults and maintain a certain level of diagnostic accuracy. This demonstrates that FedAdv_ID not only eliminates fault information discrepancies among clients but, more importantly, captures useful fault information from clients. It also effectively leverages unique fault information within imbalanced fault categories, thereby enhancing the fault diagnosis capability of the federated model. In addition, compared to direct aggregation by FedAvg, the adversarial loss function with regularization ensures consistency in the optimization direction of client parameters, reducing distribution differences between fault features. The aggregation strategy based on fault information differences fully considers the quality of client data, ensuring that useful information from low-quality clients is effectively integrated into the federated model. This improves the utilization of useful information, allowing FedAdv_ID to achieve better fault diagnosis results in more complex data scenarios compared to other methods.

### 4.3. Description of SMU Test Bench and Dataset

This section aims to evaluate the effectiveness of the proposed method on a multi-sensor, multi-condition dataset. This experiment utilizes data collected from the motor fault test bench at SMU. In multi-sensor signal fault diagnosis tasks, each data channel captures different characteristic manifestations of the same fault, providing rich and diverse data. This multidimensional information allows for a more comprehensive assessment of the motor’s operational state, offering a more accurate basis for fault diagnosis. However, the richness of this information also exacerbates the disparity in fault information across clients, potentially impacting the diagnostic performance of the federated model. To address this challenge, this study employs the SMU motor dataset to validate the proposed method’s effectiveness in eliminating fault information disparities. Special attention is given to its diagnostic accuracy in complex scenarios, such as multiple operational conditions and missing fault categories. Through this experiment, we aim to demonstrate the robustness and adaptability of the proposed method in handling high-dimensional, heterogeneous data, thereby providing stronger support for its application in real industrial environments.

**Figure 8:**
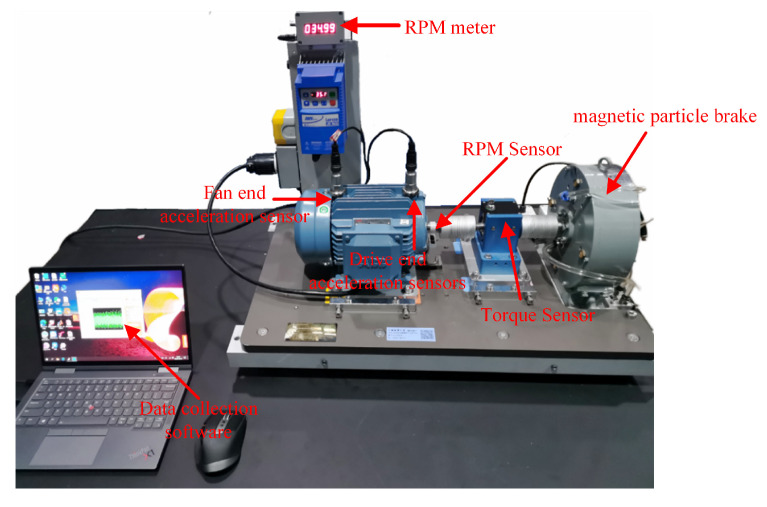
SMU motor fault test bench.

The SMU motor fault test bench is shown in Figure 8. This platform comprises a 2.2 kW three-phase asynchronous motor manufactured by ABB Group (The motor model is M2QA), a motor controller, a torque sensor, a magnetic powder brake, and data acquisition software. Various fault conditions can be simulated by replacing motors with different defects.

This platform simultaneously monitors physical quantities across seven channels, including vibration signals, three-phase currents (U, V, W), torque, and rotational speed. Vibration signals are captured via accelerometers installed at both the fan and drive ends; three-phase currents are monitored by integrating current clamps with the input wiring; and torque and rotational speed are measured using dedicated sensors. The loading device can provide a load range of 0–10 Nm.

**Figure 9:**
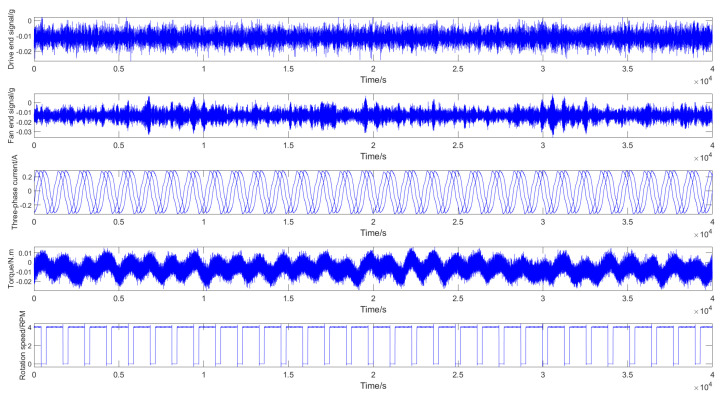
Time–domain waveform of the normal signal of SMU motor fault experiment platform.

As shown in Table 7, the platform can simulate a diverse array of fault types. Figure 9 presents the time–domain waveforms of various monitoring signals under normal working conditions. This comprehensive data acquisition and fault simulation capability provides an ideal experimental environment for in-depth research on motor fault diagnosis.

### 4.4. Complex Experimental Scenario Design for SMU Dataset

In this section, we focus on vibration signals sampled at 25.6 kHz. A sliding window technique segments the original vibration signals and facilitates practical data analysis. The window length is set to 100 data points with a step size of 50 points. This segmentation approach ensures data continuity while providing sufficient overlap to capture the dynamic characteristics of the signals.

Table 8 provides a detailed list of the fault types considered in this study, along with their corresponding labels. The selection of these fault types aims to encompass a wide range of anomalous conditions commonly encountered in motor operations, thereby offering a comprehensive testing basis for fault diagnosis algorithms. Through this systematic approach to data processing and fault classification, we aim to establish a robust experimental framework to thoroughly validate the effectiveness and generalization capability of the proposed method.

To thoroughly evaluate the effectiveness and robustness of the proposed method, particularly its performance under extreme scenarios, we designed two sets of experiments that encompass a broader range of working conditions. These experiments are intended to simulate the complex and variable operating conditions encountered in real industrial environments. Table 9 provides a detailed overview of the parameters and settings for these two experimental sets.

The model structure and hyperparameters of the comparison methods involved and the proposed method are shown in Table 10.

### 4.5. Experimental Results Analysis for the SMU Dataset

The SMU motor fault tester uses a multi-sensor system that collects data from multiple channels, including vibration signals, current signals, and rotational speed, as the basis for fault diagnosis. Compared to the single-channel sensor data from the CWRU dataset, this multidimensional data collection approach provides richer and more diverse fault information. The results of Experiment 1 show that even when processing such complex multi-channel fault data, the FedADV_ID method exhibits superior diagnostic performance. FedADV_ID shows excellent fault diagnosis accuracy compared to other methods, improving by 37.78% over the DNN method and reducing the error rate by 46.55% over the second-best method. This significant performance improvement can be attributed to the rich features of multi-channel fault data, which enables the model to learn fault characteristics from various physical quantities. Even if the vibration signals do not show any anomalies at a particular time, changes in other physical quantities can be detected promptly, leading to accurate fault diagnosis. Compared to Experiment 1, the increased complexity of working conditions in Experiment 2 led to a decline in fault diagnosis accuracy across all comparison models. However, FedAdv_ID exhibited the smallest decline in accuracy, indicating that its adversarial training mechanism based on fault feature consistency measurement effectively mitigates data distribution discrepancies between clients caused by changes in working conditions. Furthermore, a comparison between FedRL_F1 and WAFedL demonstrates that merely adding a single model parameter regularization term can only eliminate inconsistencies between models, but it fails to fundamentally address the differences between fault features. This results in the continuous amplification of model discrepancies in subsequent federated processes. Moreover, while dynamic weighting can reduce the impact of low-quality clients on the global model when the data quality of clients varies, the differences between features still interfere with the generalization capability of the federated model (Table 11).

In addition, the advantage of the FedADV_ID method is further demonstrated by its unique consistency evaluation mechanism. By constructing evaluation metrics, this method can quantify the degree of deviation between local client-specific fault information and the average fault information at the federated center. Using the federated adversarial framework, FedADV_ID eliminates this information discrepancy through interactive adversarial training, ensuring that the global model remains consistent with the optimization directions of different clients, further improving the accuracy of fault diagnosis.

Experiments 3 and 4 investigated the performance of different comparative models under multiple working conditions where certain fault types were missing. In Experiment 3, compared to Experiment 1, the absence of specific fault types under the same conditions further exacerbated the statistical distribution heterogeneity. Due to the inherent feature drift in fault data, the DNN model exhibited significant negative transfer under these conditions, rendering it almost ineffective in both experimental scenarios. Additionally, the DNN model lacks the information fusion capabilities of federated learning, resulting in a trained model that fails to capture missing fault features. These two factors together significantly compromised the diagnostic accuracy of the DNN model (Table 12).

In contrast, FedAvg addressed the issue of missing faults through collaborative information complementation, leveraging the feature extraction capabilities of the global model to compensate for its shortcomings in diagnosing unobserved fault types. Compared to DNN, this method improved fault diagnosis accuracy by 12.25%, but the fault feature differences caused by varying working conditions still limited its diagnostic performance. FedRL_F1, compared to FedAvg, further enhanced the collaborative training of the global model by incorporating federated learning and mitigated model drift through parameter regularization, minimizing the bias between local and global models and reducing the adverse effects of model heterogeneity. Similarly, Fed_BN reduced feature scale differences across different clients by normalizing the features, aiming to mitigate the impact of heterogeneity. Therefore, although the strategies of FedRL_F1 and Fed_BN for diagnosing missing fault types still focus on optimizing the global model, they outperform FedAvg in terms of accuracy.

On the other hand, WAFedL constructed a globally optimal model through the federated center’s loss function to support local optimization across clients. However, its dynamic weighted aggregation approach only assigns weights to local models based on validation set performance without fully considering the impact of client data distribution differences on the global model, leading to a significant drop in diagnostic accuracy in Experiment 4 compared to Experiment 3.

**Figure 10:**
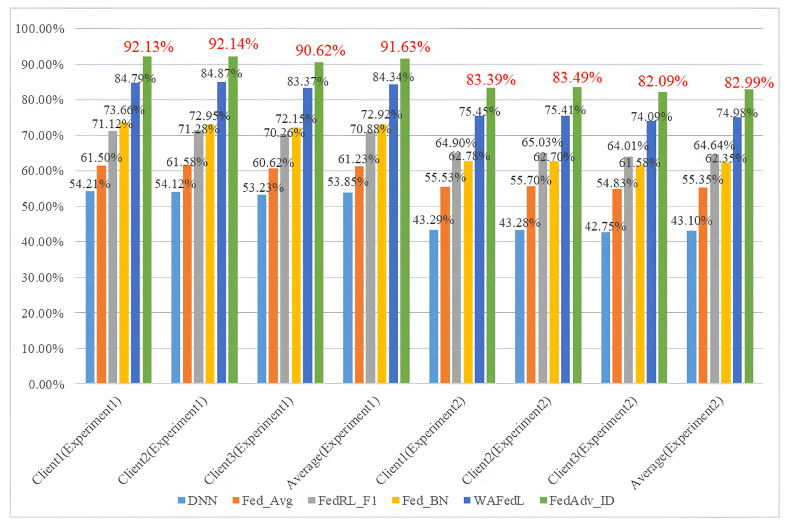
Experimental accuracy of SMU motor fault diagnosis.

The proposed FedADV_ID method eliminates client discrepancies caused by changes in working conditions at the fault information level by minimizing the difference between local fault information and average fault information. It can learn features of all known fault types in the federated center’s average fault information, compensating for the shortcomings in diagnosing missing fault types. This ensures that each client has the capability to diagnose fault types that have not been observed locally. Although the diagnostic results of the proposed method in Experiment 4 declined compared to other scenarios; its decline was the smallest, and its diagnostic accuracy was the highest among all methods, further demonstrating the superiority of the proposed method. Figure 10 visually showcases the accuracy advantage of the proposed method through experiments on a multi-channel dataset. By improving these methods, the proposed method not only ensures comprehensive diagnostic capability under different client environments but also significantly enhances overall fault diagnosis accuracy.

To further validate the superiority of the proposed algorithm across various experimental scenarios, the STAC statistical platform was employed to assess the experimental results under different experimental designs [44]. Under normal distribution testing, the results obtained by all algorithms within the same experimental scheme followed a normal distribution, aligning with actual fault diagnosis outcomes. Diverse fault diagnosis algorithms consistently yielded results displaying the classification label with the highest probability across different experimental designs, thus conforming to the normality of the results. Among numerous non-parametric test outcomes, FedAdv_ID exhibited the best overall performance and highest score compared to all other algorithms across various experimental settings. The consistent ranking of the remaining comparison algorithms with the actual experimental results further underscores the superiority of the proposed method relative to other approaches.

## 5. Conclusions and Future Work

FL has been widely adopted to address the challenges of insufficient fault samples within individual clients and the limited generalizability of fault models. However, due to the dynamic changes in motor operating conditions across different clients, significant disparities in the statistical distribution of fault data arise. These disparities limit the generalization capability of the aggregated global model and induce negative transfer phenomena in local clients, ultimately reducing fault diagnosis accuracy. This study proposes a novel method, FedAdv_ID, which begins by constructing a consistency evaluation metric to assess the degree of deviation of local fault information relative to the average fault information at the federated center. Subsequently, within an adversarial federated framework, interactive adversarial training between local clients and the federated center is employed to reduce information discrepancies and mitigate the drift in local fault information caused by statistical distribution heterogeneity. This process enhances the generalization capability of the global model. Finally, an information discrepancy-driven federated aggregation strategy is designed at the federated center to construct an optimal global model by taking into account the degree of information deviation across different clients, thereby maximizing the support for local model optimization. Experimental validation using both the CWRU single-channel dataset and the SMU multi-channel dataset demonstrates that the proposed method significantly outperforms existing approaches in terms of diagnostic accuracy.

The proposed method effectively addresses the challenge of ensuring fault diagnosis effectiveness under conditions of multiple operational modes and insufficient fault types. By utilizing benchmark and private datasets, four experimental scenarios with different operational conditions and insufficient fault types were designed to validate the effectiveness of the FedAdv_ID method. Although the proposed method demonstrates excellent performance in handling fault diagnosis across clients with varying fault information distributions due to differences in operational conditions or fault types, there are still some unresolved limitations. Specifically, when fault data collected by online monitoring equipment is subjected to network attacks, resulting in high noise levels, the inconsistency in fault information among clients increases significantly. In such cases, the proposed method lacks an effective mechanism to filter useful information from high-noise data, thereby impacting the model aggregation effect and the personalization strategy for updating the federated model. Future research could build on the current method by designing a personalized information enhancement mechanism to improve the model aggregation effect within the federated framework and enhance the processing of useful fault information.

## Figures and Tables

**Table 1:** Fault types and corresponding labels.

Fault Type	Fault Size (Inch)	Label
Ball fault	0.007	0
Inner race fault	0.007	1
Outer race fault	0.007	2
Normal	0	3

**Table 2:** Experimental scenario design for the CWRU dataset.

Experimental Scenarios	Clients	Motor Working Conditions	Fault Type	Quantity of Label Data
Experiment 1	client 1	0 HP/1 HP	0/1/2/3	4 × 120
	client 2	1 HP/2 HP	0/1/2/3	4 × 120
	client 3	2 HP/3 HP	0/1/2/3	4 × 120
Experiment 2	client 1	0 HP/1 HP/2 HP	0/1/2/3	4 × 120
	client 2	1 HP/2 HP/3 HP	0/1/2/3	4 × 120
	client 3	0 HP/2 HP/3 HP	0/1/2/3	4 × 120
Experiment 3	client 1	0 HP/1 HP	0/1/2	4 × 120
	client 2	1 HP/2 HP	0/2/3	4 × 120
	client 3	2 HP/3 HP	1/2/3	4 × 120
Experiment 4	client 1	0 HP/1 HP/2 HP	0/1/2	4 × 120
	client 2	1 HP/2 HP/3 HP	0/2/3	4 × 120
	client 3	0 HP/2 HP/3 HP	1/2/3	4 × 120

**Table 3:** Comparison methods and their descriptions.

Comparison Methods	Related Description
DNN	Traditional DL fault diagnosis methods
FedAvg [14]	Traditional FL approach
FedRL_F1 [29]	A FL approach based on F1 regularization
Fed_BN [27]	FL approach to incorporate BN layers in fault diagnosis model
WAFedL [32]	A FL approach to adaptive weight aggregation
FedAdv_ID	An information discrepancy-driven federated adversarial learning approach

**Table 4:** Model structure and hyperparameters.

Hidden Layers and Neurons	Learning Rate	Batch Size	Optimizer	Training Stopping Threshold
100/600/400/100/4	0.001	128	Adam	$10^{-4}$

**Table 5:** Comparative fault diagnosis results for experiments 1 and 2.

Experiments	Clients	DNN	FedAvg	FedRL_F1	Fed_BN	WAFedL	FedAdv_ID
Experiment 1	Client 1	55.20%	61.66%	73.94%	76.71%	83.53%	93.45%
	Client 2	53.59%	61.74%	75.79%	74.88%	81.35%	93.14%
	Client 3	52.61%	61.22%	75.87%	73.11%	83.82%	92.69%
	Average	53.80%	61.54%	75.20%	74.90%	82.90%	93.09%
Experiment 2	Client 1	43.61%	52.62%	62.67%	63.01%	73.94%	86.76%
	Client 2	45.27%	55.67%	62.42%	63.95%	75.79%	85.93%
	Client 3	46.35%	53.72%	61.02%	61.78%	75.87%	85.51%
	Average	45.07%	54.00%	62.03%	62.91%	75.20%	86.06%

**Table 6:** Fault diagnosis results for Experiments 3 and 4.

Experiments	Clients	DNN	FedAvg	FedRL_F1	Fed_BN	WAFedL	FedAdv_ID
Experiment 3	Client 1	39.51%	46.44%	56.84%	59.22%	67.29%	82.51%
	Client 2	39.65%	46.44%	56.67%	59.24%	66.99%	81.77%
	Client 3	39.52%	45.88%	56.08%	58.35%	66.49%	81.56%
	Average	39.56%	46.25%	56.53%	58.93%	66.92%	81.94%
Experiment 4	Client 1	33.99%	40.80%	55.74%	54.08%	62.66%	77.17%
	Client 2	33.88%	40.80%	55.71%	54.14%	62.62%	77.22%
	Client 3	33.63%	40.53%	54.81%	53.29%	62.19%	76.09%
	Average	33.83%	40.71%	55.42%	53.83%	62.49%	76.82%

**Table 7:** SMMU Motor Data Fault Type Description.

Fault Type	Load (N/m)	Sampling Frequency	Fault Degree
Normal	0/4/6/10	25,600	0
Inner race fault			0.5 mm
Outer race fault			0.3 mm
Phase fault			Lack of V-phase
Winding short circuit fault			Winding short circuit 10%
Winding short circuit fault			Winding short circuit 5%
Shaft bending fault			0.3 mm
Rotor unbalance fault			4 g
Parallel misalignment fault			0.25 mm
Broken bar fault			Broken two bars

**Table 8:** Experimental fault types and corresponding labels.

Fault Type	Fault Degree	Label
Fan end bearing inner race fault	0.05 mm	0
Drive end bearing outer race fault	0.05 mm	1
Bent shaft	0.1 mm	2
Broken bar fault	two	3

**Table 9:** Design of complex experimental scenarios.

Experiments	Clients	Working Conditions (N·m)	Fault Type	Quantity of Labeled Data
Experiment 1	Client 1	0/6	0/1/2/3	4 × 100
	Client 2	0/10	0/1/2/3	4 × 100
	Client 3	6/10	0/1/2/3	4 × 100
Experiment 2	Client 1	0/4/6	0/1/2/3	4 × 100
	Client 2	4/6/10	0/1/2/3	4 × 100
	Client 3	0/6/10	0/1/2/3	4 × 100
Experiment 3	Client 1	0/6	0/1/2	4 × 100
	Client 2	0/10	0/1/3	4 × 100
	Client 3	6/10	1/2/3	4 × 100
Experiment 4	Client 1	0/4/6	0/1/2	4 × 100
	Client 2	4/6/10	0/1/3	4 × 100
	Client 3	0/6/10	1/2/3	4 × 100

**Table 10:** Structure and hyperparameters of all models.

Hidden Layers and Neurons	Learning Rate	Batch Size	Optimizer	Training Stopping Thresholds
100/1200/600/400/4	0.001	128	Adam	$10^{-4}$

**Table 11:** Client diagnostic accuracy of different methods in Experiments 1 and 2.

Experiments	Clients	DNN	FedAvg	FedRL_F1	Fed_BN	WAFedL	FedAdv_ID
Experiment 1	Client 1	54.21%	61.50%	71.20%	73.66%	84.79%	92.13%
	Client 2	54.12%	61.58%	71.20%	72.95%	84.87%	92.14%
	Client 3	53.23%	60.62%	70.26%	72.15%	83.37%	90.62%
	Average	53.85%	61.54%	70.88%	72.92%	84.34%	91.63%
Experiment 2	Client 1	41.43%	49.15%	61.53%	65.37%	73.97%	85.83%
	Client 2	41.87%	48.93%	60.86%	66.05%	74.62%	86.47%
	Client 3	42.56%	51.24%	62.31%	65.79%	75.08%	85.12%
	Average	41.95%	49.77%	61.56%	65.74%	74.55%	85.81%

**Table 12:** Client diagnostic accuracy of different methods in Experiments 3 and 4.

Experiments	Clients	DNN	FedAvg	FedRL_F1	Fed_BN	WAFedL	FedAdv_ID
Experiment 3	Client 1	43.29%	55.53%	64.90%	62.78%	75.45%	83.39%
	Client 2	43.28%	55.70%	65.03%	62.70%	75.41%	83.49%
	Client 3	42.75%	54.83%	64.01%	61.58%	74.09%	82.09%
	Average	43.10%	55.35%	64.64%	62.35%	74.98%	82.99%
Experiment 4	Client 1	32.47%	42.61%	51.55%	55.81%	63.76%	79.49%
	Client 2	33.36%	41.85%	50.91%	56.27%	64.25%	78.22%
	Client 3	32.24%	42.17%	52.74%	56.38%	63.71%	77.65%
	Average	32.69%	42.21%	51.76%	56.15%	63.91%	78.45%

## Data Availability

The data involved in this article have been presented in this article.
